# Application of spectroscopy in herbal metabolomics

**DOI:** 10.1186/2008-2231-20-91

**Published:** 2012-12-12

**Authors:** Soodabeh Saeidnia, Ahmad Reza Gohari

**Affiliations:** 1 Medicinal Plants Research Center, Faculty of Pharmacy, Tehran University of Medical Sciences, PO Box 14155-6451, Tehran, Iran

## 

Metabolomics is defined as “systematic study of the unique chemical fingerprints that specific cellular processes leave behind” [1]. Metabolom includes the final metabolites of a cell, or biological organisms [2]. Herbal metabolomics is unbiased, high-performance and specific analysis of highly complex mixtures of plant extracts. This overall gain for metabolom analysis is at first a result of recent advances in mass spectrometry (MS). Nowadays, about 50,000 herbal compounds have been extracted and identified from plants. It is predicted that the final number would reach 200,000. That’s why metabolomics is a considerable challenge for scientists [3]. Herbal metabolom is very diverse and includes fat-soluble compounds, polar macromolecules, basic and acidic ions, combinations of the constituents, highly stable and oxidative susceptible natural products. Herbal metabolom extraction methods and measurement tools should be selected carefully [4].

It should be memorized that single techniques can be never analyzed to execute a trustable determination. Applying quick scanned time-of-flight (TOF) mass spectrometry *via* gas chromatography (GC) resulted in the numbers of detectable metabolites to 500-1000 in total extracts. Another attractive method would be direct injection of herbal extract to ultra-high-resolution Fourier Transform Ion Cyclotron Mass Spectroscopy, which is able to produce a significant fingerprint of metabolom [5]. Compounds finger printing *via* NMR is a quick, easy and effective tool to distinguish between various compounds of a natural product group, which is attributed to detect of very important regions of a spectrum. Among the isotopes, ^1^H proton is the most sensitive magnetic atom preferred for supplying metabolic profile in most natural compounds. Another appropriate isotope is ^19^F which has a compble sensitivity to proton and applied in fluorinated plant tissues. Furthermore, ^31^P isotope conduces to profile phosphate esters that found frequently in herbal extracts. ^13^C technique is frequently used for mapping of the amino acids, carbohydrates, lipids and organic acids in herbal extracts. The above mentioned spectra may contribute to find differences in metabolite concentrations. These achievements determine the plant metabolic phenotype but it does not accomplish a complete analyze of herbal metabolite [6]. NMR identification of the main components in the essential oils or herbal extracts clarifies the possible chemotaxonomic differences between plant species. As an example, identification of limonene-10-al, one of the main chemical markers among *Dracocephalum* species, was carried out by NMR, while analysis of the volatile oils did not detect this major compound in several GC/MS studies [7]. There are many factors to criticize efficiency of MS and NMR as metabolomics techniques. For example, both two methods need tissue extraction. Some compounds should be derivated before being injected to GC/MS. For this reason, providing mass spectrum samples would be time consuming and also fragmentation peaks in mass spectrum does not imply the actual compounds. Both two techniques (NMR and Mass) produce different peaks which could be an advantage in detecting metabolites while creating complexity. However mass spectrometry is capable of detecting low amounts of metabolites, NMR spectra complete it and play an important role in wide systems analyzing. Using LC/MS and LC/NMR causes to eliminate the septing stages to refine the compounds in phytochemical analyses. Directed transaction from a column to a preserving and storing loop may consequently result in high quality peaks. Using a SPE system (solid phase extraction) between HPLC and NMR increases the sensitivity in detecting natural compounds from herbal extracts. Using the SPE cartridge would result in accumulating and condensing of sample to avoid applying great values of deuterated solvent which is necessary in LC/NMR. Today, LC-UV-SPE-NMR-MS are widely used in genobiotic metabolism studies and related researches in natural compounds [6,8].

Combination of GC/MS and LC/MS is able to realize hundreds of chemical compounds including: glycosides, alcohol glycosides, organic acids, amino acids, fatty acids and a wide range of various secondary metabolites. GC/MS and LC/MS are effectively functioned, especially in different chemical isomers like hexoses which all have an equal mass spectrum. Besides essential oils, diethyl ether extracts of the medicinal plants have been reported to analyze by this method [9,10].

Herbal metabolomics would be efficient for evaluation and quality control of traditional and folk medicine, especially large scale analysis of herbal medicines as a complementary to common quality control methods. The future innovative goal would be developing the evaluation system of medicinal plants and phytopharmaceutical products based on the analytical results. Although rapid advances in analytical techniques have been occurred, interpretation of an herbal metabolomic profile remains difficult. This is attributed to the complexity of natural product mixtures which makes the evaluation of their function incomprehensive. Optimizing the seption techniques and improving the sensitivity of NMR and MS instruments have been essentially recommended to gain higher resolution and greater accuracy. To avoid lack of reproducibility of results, following the Standard Operating Protocols for plant extraction and seption are necessary. In addition, recognition of bioinformatics, computational databases and metabolic networks needs to develop among the researchers in various fields of cultivation, biological and phytochemical evaluations of medicinal plants.

## Authors’ contributions

SS (conception and design); GAR (drafting the article and revising it critically for important intellectual content and final approval of the version to be published).
